# Left-handed metamaterial inspired by joint T-D geometry on flexible NiAl_2_O_4_ substrate

**DOI:** 10.1371/journal.pone.0199150

**Published:** 2018-06-20

**Authors:** Eistiak Ahamed, Md. Mehedi Hasan, Mohammad Rashed Iqbal Faruque, Mohd Fais Bin Mansor, Sabirin Abdullah, Mohammad Tariqul Islam

**Affiliations:** 1 Space Science Center (ANGKASA), Universiti Kebangsaan Malaysia, UKM, Selangor, Malaysia; 2 Department of Electrical, Electronic and Systems Engineering, Universiti Kebangsan Malaysia, UKM, Bangi, Selangor, Malaysia; Ludwig-Maximilians-Universitat Munchen, GERMANY

## Abstract

In this paper, we introduce a new compact left-handed tunable metamaterial structure, inspired by a joint T-D shape geometry on a flexible NiAl_2_O_4_ substrate. The designed metamaterial exhibits an extra-large negative refractive index bandwidth of 6.34 GHz, with an operating frequency range from 4 to 18 GHz. We demonstrate the effects of substrate material thickness on the effective properties of metamaterial using two substrate materials: 1) flame retardant 4 and 2) flexible nickel aluminate. A finite integration technique based on the Computer Simulation Technology Microwave Studio electromagnetic simulator was used for our design, simulation, and investigation. A finite element method based on an HFSS (High Frequency Structure Simulator) electromagnetic simulator is also used to simulate results, perform verifications, and compare the measured results. The simulated resonance peaks occurred at 6.42 GHz (C-band), 9.32 GHz (X-band), and 16.90 GHz (Ku-band), while the measured resonance peaks occurred at 6.60 GHz (C-band), 9.16 GHz (X-band) and 17.28 GHz (Ku-band). The metamaterial structure exhibited biaxial tunable properties by changing the electromagnetic wave propagation in the y and z directions and the left-handed characteristics at 11.35 GHz and 13.50 GHz.

## Introduction

A metamaterial is an artificial composite periodic material with unusual properties that are unavailable in nature. Metamaterials have greatly interested the scientific research community because of their unique characteristics, such as negative refraction, perfect absorption, magnetism, sub-wavelength focusing, varying chiralities and so on. Because of their cellular architecture (rather than chemical composition), metamaterials exhibit unusual electromagnetic properties, and the materials can control electromagnetic wave beams in predictable ways. Because of these peculiar electromagnetic properties, metamaterials have unique potentials for various applications, such as antenna applications, power absorption, sensing, terahertz applications, energy harvesting, super lens applications, etc. In 1967, V. G. Veselago [1] recommended a concept of reverse material behaviour, but the idea attracted little interest in the scientific community, because such a material was not available. This was true before 1996, when Pendry et al [2] presented a thin wire formation that displayed negative permittivity and a split ring resonator (SRR) that exhibited negative permeability. In 2000, Smith et al. [3] presented a design that simultaneously exhibited negative permittivity and negative permeability, and conducted a microwave examination to assess its rare electromagnetic properties. Recently, metamaterials having wide negative refractive index bandwidths have been developed. Various alphabetical shapes have been proposed for metamaterial applications. For instance, Hasan et al. [4] produced 10 × 10 mm^2^ S-shaped metamaterial for X-band operation in the microwave region for satellite communication applications. Hossain et al. [5] proposed a double G-shaped left-handed metamaterial which was applicable for S and C band in microwave regime. Hasan et al. [6] proposed a left-handed biaxial metamaterial that showed negative index properties from 8.40 to 12.07 GHz in the z-direction and from 11.68 to 14.37 GHz in the y-direction of electromagnetic wave propagation. Turkmen et al. [7] proposed a multi-ring U-shaped metamaterial that was applicable to the C- and X-bands in the microwave region. Multi-band functionality was created, but the metamaterial did not display left-handed characteristics. Benosman et al. [8] recommended a dual S-shaped metamaterial that showed a negative refractive index in Ku band. Hasan et al. [9] proposed a square I-shaped metamaterial that for S-, C- and Ku-band applications. Zhou et al. [10] proposed a 15 × 15 mm^2^ S shaped metamaterial applicable for the X- and Ku-bands, but its effective medium ratio was less than 4. Riwan et al. [11] proposed a left-handed F-shaped metamaterial for microwave applications. Their designed structure exhibited a 2.3 GHz negative refractive index bandwidth. Dhouibi et al. [12] suggested a single negative Z-shaped metamaterial that was examined by one-axis wave propagation for C-band applications. However, all of them proposed their designs for only one axis of electromagnetic wave propagation, and their proposed negative refractive index were also quite low.

By tuning the properties of a component (or by simply adjusting the geometries of a metamaterial structure), it is possible to modify the general properties of artificial materials. Material tuning properties are critical for many applications, like the flexible control of wave propagation, tunable lenses, sensor technology, etc. To tune the resonance of an SRR, Aydin et al. [13, 14] changed the geometrical parameters of a material. As a result, the effective electromagnetic properties were unusual, with values close to the resonance frequencies created by the implanted SRR structure. Zharov et al. [15] represented a nonlinear dielectric substrate resonance structure with properties that relied on external fields. Lapine et al. [16] presented a tuning metamaterial that exhibited a negative response for effective permeability, depending on the external magnetic fields and applying external fields. The metamaterial band gap was studied by varing the resistance and capacitance properties. Reynet et al. [17] presented a metamaterial that could be tuned using conducting coils containing a vericap diode and a resonance controlled by the applied field. A similar theory was presented by Gil et al. [18], who proposed a tunable micro strip notch filter that depended on varactor loaded SRRs. Additionally, when modeling the effective properties of a metamaterial, it should be ensured that the resonance wavelength is high enough to be compared with the lattice dimensions of the unit cell. However, in the terahertz and optical regions, it is critical that the dimensions be adjustable. In a metamaterial, the metal strip is typically permanent, and is difficult to modify at high frequencies the split gaps are too minor to tune accurately.

In this paper, a new left-handed metamaterial structure with a modified substrate is proposed. The proposed metamaterial exhibits resonance frequencies in the C-band, X-band and Ku-band in the microwave regime. Two different types of substrate are used to introduce this new metamaterial design: a conventional flame retardant 4 (FR-4) substrate, and a nickel aluminate (NiAl_2_O_4_) substrate. The conventional FR-4 substrate is used to verify the newly developed NiAl_2_O_4_ substrate. The proposed structure is analyzed in both directions (z- and y-direction) using electromagnetic wave propagation. With respect to z-axis wave propagation, the proposed NiAl_2_O_4_-substrate-based metamaterial exhibited almost a left-handed refractive index relative to the proposed FR-4-based metamaterial design. The proposed structure can be characterized as a biaxial metamaterial that shows left-handedness in both directions and for both substrates. We varied the thicknesses of the two substrates and examined how these changes affected the effective parameters and resonances. In this way, we obtained a polynomial equation that can help us understand how to use thickness variations to change the resonances and effective parameters of the material. We demonstrated that the metamaterial properties can be successfully tuned by varying the substrate thickness. Our analytical and numerical results were in agreement. To produce tunable metamaterials, the thickness of the material can be used as a controller for the design of a tunable dielectric substrate. It shows tuning properties when the direction of electromagnetic wave propagation changes and bring variation on its substrate thickness.

## Flexible substrate formation and design of metamaterial structure

**Fig 1:**
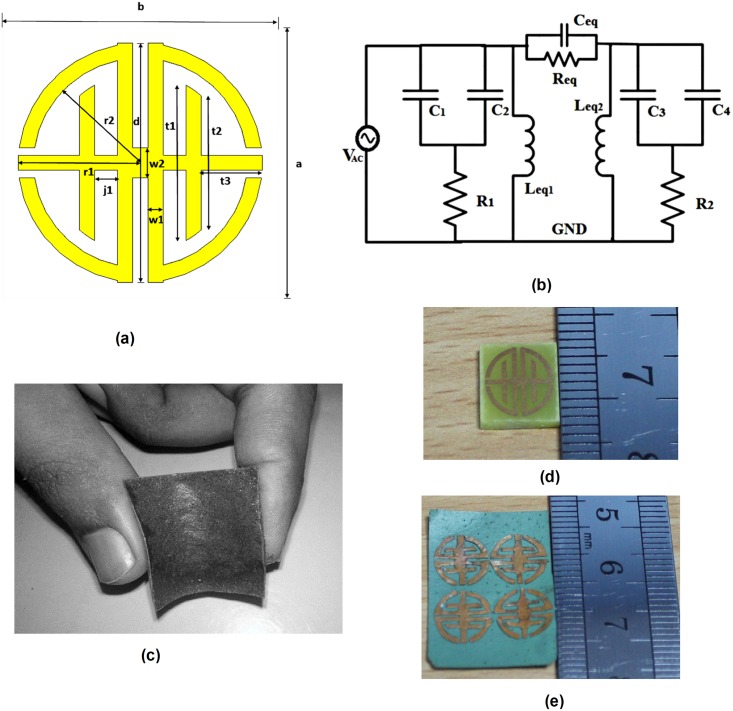
Metamaterial joint T-D geometry, equivalent circuit and fabrication. (a) Proposed metamaterial unit cell structure, (b) equivalent circuit [19], (c) Flexible Nickel aluminate material (NiAl_2_O_4_); fabricated proposed design on (d) FR-4 substrate and (e) 2×2 array prototype on flexible NiAl_2_O_4_ substrate.

The proposed tunable metamaterial unit cell structure is composed of a joint T-D shaped SRR and its schematic view is shown in Fig 1(a). The structure was designed with T- and D-shaped parts. One part is symmetrical with another part, and the two are connected by an electrical slab. The designed T-shaped part has horizontal and vertical components. The horizontal part is indicated by t_3_, while the vertical part is represented by t_1_ and t_2_. The D-shaped part has also two components: a half-circular path and a vertical path. The half-circular path is indicated by r_1_ and r_2_, while the vertical path is indicated by d. The T- and D-shaped parts are joined by an electrical slab, indicated by j_1_. The designed joint T-D shaped metamaterial structure is fabricated on two substrates: FR-4 and NiAl_2_O_4_. The proposed design is coated with copper on the front side of the FR-4 substrate. An FR-4 substrate is chosen with a permittivity of ɛ_r_ = 4.3 and loss tangent of δ = 0.02. The selected FR-4 substrate is a 9 × 9 mm^2^ square with a thickness (t_4_) of 1.6 mm for the proposed metamaterial unit cell. The copper coating thickness is 0.035 mm, with an electrical conductivity of σ = 5.8 × 107 S/m. The fabricated unit cell structure of the FR-4 substrate is shown in Fig 1(d).

In recent year, several flexible substrates have been used: ceramic substrates, NiAl_2_O_4_ substrates, manganese zinc ferrite substrates, etc. In this study, our flexible NiAl_2_O_4_ composite samples (shown in Fig 1(c)) were created using a combination of two main raw materials: aluminium nitrate nonahydrate Al(NO_3_)_3_.9H_2_O and nickel nitrate hexahydrate Ni(NO_3_)_2_.6H_2_O. The stoichiometry volumes of the nitrate powders were used to identify a molar ratio of 0.42(nickel):0.58(aluminium nitrate), termed Ni_42_. To prepare the samples, these were liquefied into purified water with citric acid as a chelating agent. A glutinous solution is made that exhibits light green and transparent characteristics. The sample is prepared at lower temperatures by a suitable citrate salt-gel method, which has an admirable mechanism over the stoichiometry and easier dopant outline. The prepared solution was heated to 90 °C and stirred continuously for approximately 4 hours. The water was completely evaporated, leaving a greenish gel. The earlier gel was placed into an alumina crucible furnace (model: 56000) at 150 °C to complete the chemical reaction, and to produce a fine powder. Then, the precursor was ground appropriated and calcinated for 1 hour at 450°C. Additionally, polyvinyl acetate (PVA) glue was produced by adding PVA, with distilled water as a binder. The NiAl_2_O_4_ substrate was obtained by adding the synthesized powder to the PVA solution at a ratio of 1 g: 10 mL and stirring properly. The loss tangent and dielectric constant were measured over a frequency range of 1–8 GHz at room temperature. We used a DAK 200 MHz to 20 GHz dielectric measurement kit to obtain relative permittivity (4.97) and dielectric loss tangent (0.000514) of the samples. The prepared substrate is 20 × 20 mm^2^ in size and 0.60 mm thick. In Fig 1(e), a prepared 2 × 2 array prototype is shown, where copper tape is used to make the proposed design on top of the substrate.

The electromagnetic field is mainly delivered to the structure to get different radiation properties and displays some rare characteristics. The parameters and dimensions are identified in the Table 1. Table 1 specifies that ‘a’ is the substrate length and ‘b’ is the substrate width. The D- alphabet half circular shape’s outer radius is r_1_ and inner radius is r_2_. The metal strip width is w_1_ and the electric slab length that connected two symmetrical joint T-D shaped is w_2_. T shape’s head is indicated by t_1_ and t_2_ and the joint point length of T and D shape is j_1_.

**Table 1:** Design parameter of the proposed flexible metamaterial structure.

Parameters	Dimension (mm)	Parameters	Dimension(mm)
a	9.00	w_1_	0.50
b	9.00	w_2_	1.00
d	8.00	t_1_	5.20
r_1_	4.00	t_2_	4.48
r_2_	3.50	t_3_	2.00
j_1_	0.75	t_4_	1.60

## Methodology

We used numerical methods to extract the scattering parameters and identify the resonance frequencies of the proposed structure. Our numerical methods used both a finite integration technique (FIT) based on the electromagnetic simulator computer simulation technique (CST) Microwave Studio and a finite element method (FEM) based on a high-frequency structure simulator (HFSS). These scattering parameters were used to retrieve the effective refractive index (η_r_), effective permittivity (μ_r_) and effective permeability (ε_r_) of the proposed structure. For z-axis wave operations, the prototypes were examined by placing them between two waveguide ports, and by exciting them with an electromagnetic force in the positive direction of the z axis. To excite negative magnetic and electric responses, the magnetic and electric fields were polarized by incident electromagnetic waves, which were defined along the y-axis and x-axis, respectively. The simulation was executed for the frequency range from 4 to 18 GHz. Based on Nicolson–Ross–Weir (NRW) method [20–21], the following Eqs (1)–(9) are used to retrieve the effective parameters.

$$\Gamma =(\frac{{Ζ}_{0}-1}{{Ζ}_{0}+1})$$ (1)

$$Z_{0}=\sqrt{\frac{\mu_{r}}{\epsilon_{r}}}$$ (2)

$$S_{11}=\{\frac{(1-\Gamma^{2})\;Ζ\;}{1-\Gamma^{2}{Ζ}^{2}}\}$$ (3)

$$S_{21}=\{\frac{(1-{Ζ}^{2})\;\Gamma \;}{1-\Gamma^{2}{Ζ}^{2}}\}$$ (4)

Where, *Z*_0_ is the relative impedance, Γ is the reflection coefficient. Now, from *S*_11_ and *S*_21_,

$$V_{1}=(S_{21}+S_{11})$$ (5)

$$V_{2}=(S_{21}-S_{11})$$ (6)

$${Effective\;Permittivity,\;\varepsilon }_{r}=\{\frac{c}{j\pi fd}\times (\frac{1-V_{1}}{1+V_{1}})\}$$ (7)

$$\varepsilon_{r}=\;\{\frac{c}{j\pi fd}\times \frac{1-S_{21}-S_{11}}{1+S_{21}+S_{11}}\}$$

$${Effective\;Permeability,\;\mu }_{r}=\{\frac{c}{j\pi fd}\times (\frac{1-V_{2}}{1+V_{2}})\}$$ (8)

$$\mu_{r}=\{\frac{c}{j\pi fd}\times \frac{1-S_{21}+S_{11}}{1+S_{21}-S_{11}}\}$$

$${Refractive\;Index,\;\eta }_{r}=\sqrt{\varepsilon_{r}\mu_{r}}$$ (9)

$$\eta_{r}=\;\frac{c}{j\pi fd}\;\times \;\sqrt{\frac{({S_{21}-1)}^{2}-\;S_{11}^{2}}{({S_{21}+1)}^{2}-\;S_{11}^{2}}}$$

The refractive index *η*_*r*_, effective permittivity *ε*_*r*_, and effective permeability *μ*_*r*_, are retrieved from Eqs (1)–(9). The structure behaves like an LC resonant circuit. Two characteristics are responsible for generating electrical resonances in the metamaterial: one is a metal strip and another is a split gap. The metal strip is excited through a time-variable magnetic field parallel to the axis of the metal strip. The electrostatic field is induced in the split gape and inductive current in the metal strip, resulting in resonant energy interchange. The LC resonance frequency leads to a decrease in means-shifting toward a lower frequency. The inductance can be increased or decreased in a circuit by increasing or decreasing the side or length of the metal strip. Likewise, by increasing or decreasing the split capacitance can be decreased or increased, which leads to a decrease or increase in the LC resonant frequency, meaning that resonance is shifted to a lower or higher frequency. The equivalent circuit of the proposed metamaterial unit cell structure is given in Fig 1(b). From this figure, the capacitive effect of the overall structure is symbolized by C_1_, C_2_, C_3_, and C_4_, and the inductance effect is symbolized by Leq. The equivalent circuit model elements L and C create resonances that are formed by

$$f=\frac{1}{2\pi \sqrt{LC}}$$ (10)

**Fig 2:**
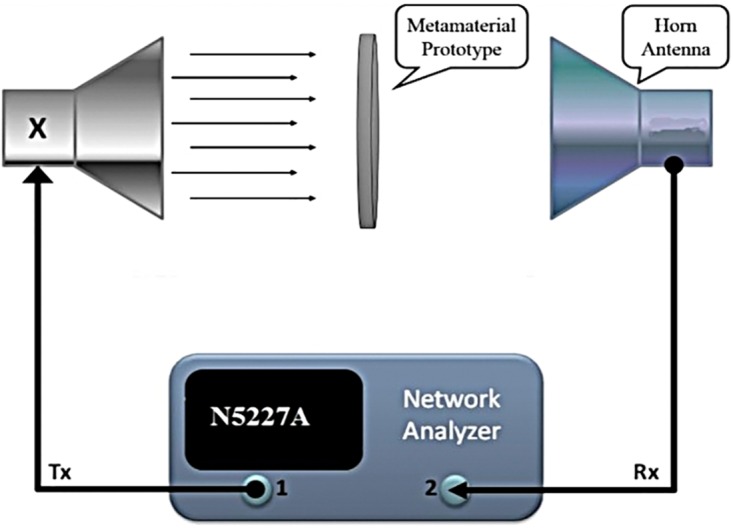
Measurement set-up of the proposed flexible metamaterial structure.

For measurement purposes, the metamaterial unit cell is positioned between two microwave waveguide ports. Three different types of wave ports are used to measure three different frequency bands: for C-band (1) A-INFOMW WG to adapter P/N: 137WCAS, for X-band (2) A-INFOMW WG to adapter P/N: 112WCAS and for Ku-band (3) A-INFOMW WG to adapter P/N: 51WCAS-CU. A vector network analyzer (VNA) model number Agilent N5227A (CA, USA) was used for measurements. The calibration process was performed by an electric calibration module (Agilent N4694-60001). A metamaterial array prototype 117 × 162 mm^2^ in size was placed between two horn antennas, as shown in the schematic in Fig 2. The incident waves travelled through the prototype as in the simulated geometry. The horn antennas were joined with an (Agilent N5227A) VNA to determine the scattering parameters of the joint T-D shaped metamaterial prototype. Additionally, an Agilent N4694-60001 was used to calibrate the N5227A VNA for accurately performing measurements.

## Results and discussion

### Using conventional substrate for metamaterial structure

The electromagnetic wave propagation is delivered in the z- and y-direction to analyze the metamaterial prototype in section 4.1.1 and 4.1.2 and section 4.1.3 represent the effect of changing substrate thickness.

#### Analyses of proposed design in z-direction

**Fig 3:**
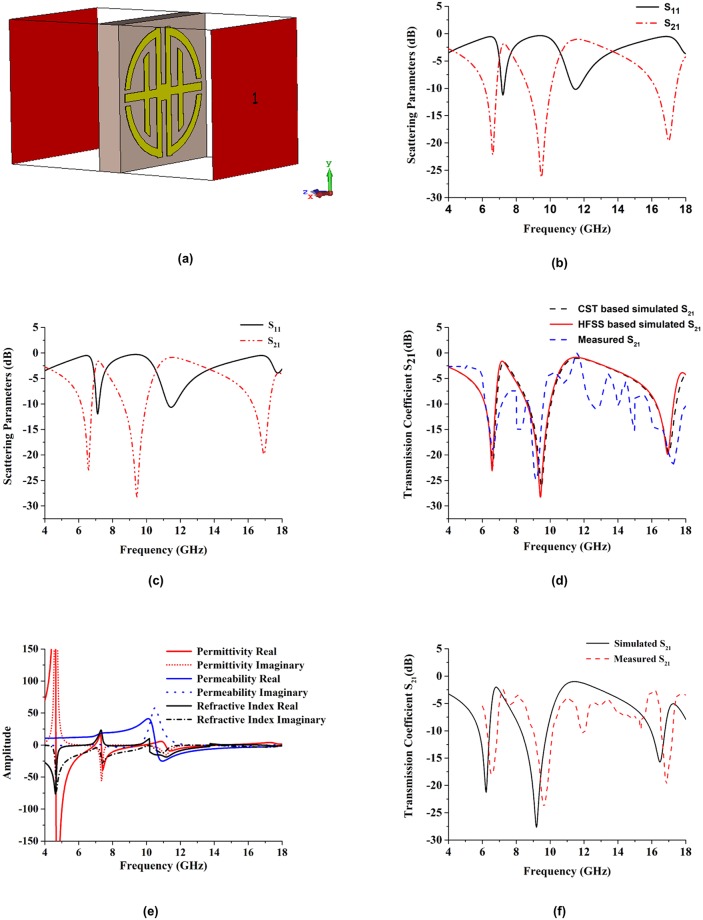
Simulation set-up with simulated and experimental results. (a) The geometry of the proposed structure in z-axis wave propagation, (b) Simulated reflection and transmission coefficients in CST, (c) Simulated reflection and transmission coefficients in HFSS, (d) The measured and simulated comparative result, (e) Amplitude of Effective parameters, (f) Results of 13 × 18 mm^2^ unit cell array refraction and transmission coefficient.

Fig 3(a) depicts the simulated geometry of the proposed metamaterial design in the z-direction of electromagnetic wave propagation. The reflection coefficient (S_11_) and transmission coefficient (S_21_) of the proposed design were analyzed by two different types of software. Both software programs produced approximately the same resonance frequency results. The simulated reflective coefficient (S_11_) and transmission coefficient (S_21_) results are shown in Fig 3(b) from the CST software base investigation. The figure shows that the resonance frequencies were 6.42 GHz (under C-band), 9.32 GHz (under X-band), and 16.90 GHz (under Ku-band). The HFSS software base results for the reflective coefficient (S_11_) and transmission coefficient (S_21_) are shown in Fig 3(c). From that figure, the resonance frequencies were 6.57 GHz (under C-band), 9.43 GHz (under X-band), and 16.95 GHz (under Ku-band). The resonance frequencies were the same for both cases. The simulated and measured transmission coefficient results for the 13 × 18 unit cell array prototype are shown in Fig 3(f). The resonance frequencies for the array prototype were 6.55 GHz (under C-band), 9.69 GHz (under X-band), and 16.83 GHz (under Ku-band). The experimental and simulated (CST- and HFSS-software-based) results for the transmission coefficient (S_21_) of the proposed structure are shown in Fig 3(d). The measured resonances were 6.60 GHz (under C-band), 9.16 GHz (under X-band), and 17.28 GHz (under Ku-band). The measured transmission coefficient (S_21_) was slightly shifted. However, almost at same resonance frequencies matched with the simulated results. Typically, that occurs because of free space measurement procedures, fabrication errors, and for improper environment. Other factors that affect measured data are the lossy cables and connectors used in measurement procedures.

The effective parameters permittivity, permeability and refractive index are shown in Fig 3(e). From that figure the effective permittivity shows negative characteristics from 4.65 GHz to 6.92 GHz (bandwidth 2.27 GHz), 7.34 GHz to 9.83 GHz (bandwidth of 2.49 GHz), and 11.11 GHz to 14.36 GHz (bandwidth of 3.25 GHz). The permeability is showed negativity from 10.55 GHz to 18 GHz. The oscillator current and applied field current is in phase when the frequency is in the lower region. However, the current lags and fails to remain in phase with the applied field at higher frequency levels. This yields negative permeability at that frequency. Negative refractive index is from 4 GHz to 6.35 GHz (bandwidth of 2.35), 7.64 GHz to 9.25 GHz (bandwidth of 1.61), 10.18 GHz to 13.73 GHz (bandwidth of 3.55 GHz), and 16.88 GHz to 18 GHz (bandwidth of 1.12 GHz). At 11.35 GHz and 13.50 GHz effective parameters are negative. Therefore, it exhibits left-handed properties at 11.35 GHz and 13.50 GHz.

**Fig 4:**
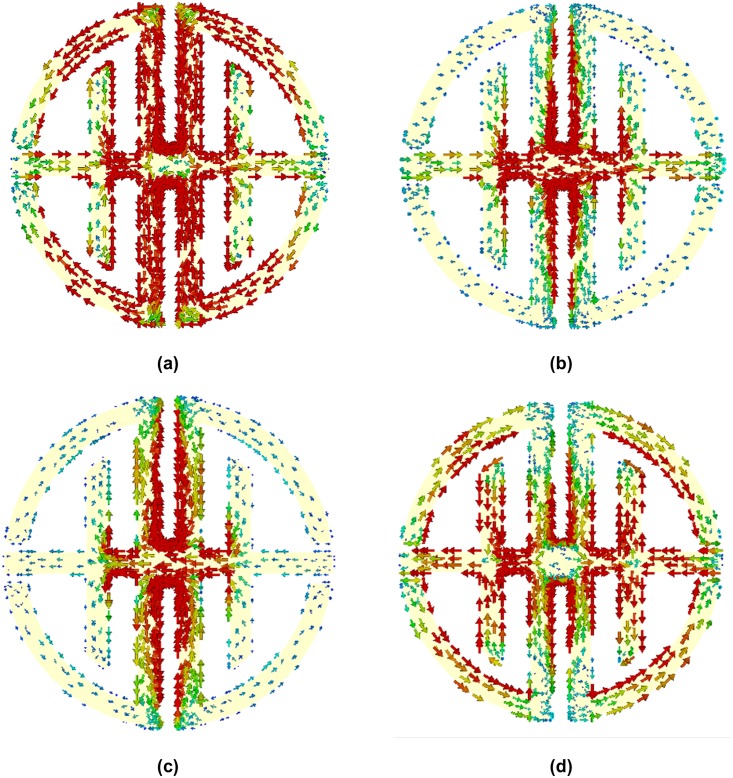
Surface current distribution at (a) 6.60 GHz, (b) 9.16 GHz, (c) 17.28 GHz and (d) 11.35 GHz.

To observe the physical phenomena of the proposed design and understand how the structure works when it is placed into an electromagnetic field region, the surface current distributions are analyzed for different frequencies. The surface current distributions of the proposed unit cell at 6.60, 9.16, 17.28, and 11.35 GHz are shown in Fig 4(a) to 4(d). The arrows represent the direction of the current distribution in the overall structure resonator, and the colors express the intensity. At 6.60 GHz in Fig 4(a), the surface current is distributed throughout the whole structure, because its measured transmission coefficient bandwidth is sufficiently large.

Although surface currents are distributed throughout the structure, they are strongly concentrated in the symmetric D-shaped vertical parts, and current flows in the opposite direction from those metal strips. This effect creates a stop band by nullifying the current. In Fig 4(c), the surface current is shown for a resonant frequency of 17.28 GHz. At that frequency, the current is weakly distributed throughout the whole structure. From Fig 4(b) and 4(d) the surface current distribution is more dance in three regions that are electric slab that connected two symmetrical joint T-D shaped, joint point of the T-D shape, and vertical component of the D shape. The surface currents flow in opposite directions in two opposing ring resonators. Therefore, a stop band is created by nullifying the current. In actuality, two conductor currents are anti-symmetric at the resonance and form a loop, which can be characterized as an equivalent magnetic dipole moment. The artificial magnetism of the structure is created in this magnetic moment, which causes the affective negative permeability of the structure.

**Fig 5:**
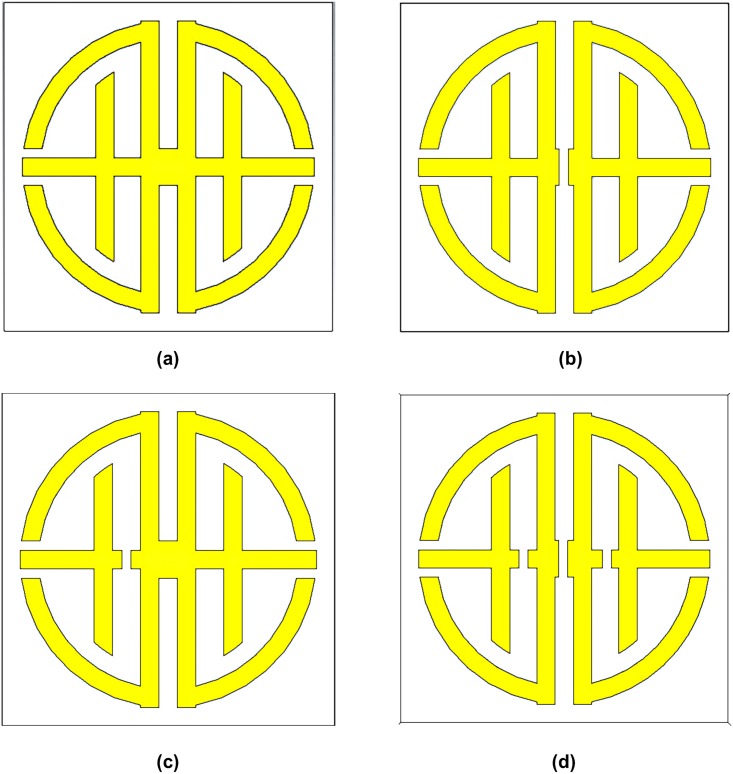
Design of metamaterial unit cell. (a) Proposed structure (b) modified structure 1 (c) modified structure 2 (d) modified structure 3.

Resonance frequency depends on various conditions such as substrate dielectric permittivity, thickness of the substrate, positioning of the substrate in the electromagnetic field, design structure on a substrate or by changing the substrate material. In this case, the resonance frequency depends on not only the substrate thickness, but also substrate itself reason for varying resonance frequency by changing its position in electromagnetic field and by using different metamaterial structure on a substrate. In this research, resonance frequency depends on metamaterial structure. It can be seen when some changes are done on the design of the proposed metamaterial structure shown in Fig 5(a). Three different modified structure of the proposed metamaterial is presented to verify the dependency of resonance frequency to the metamaterial structure. The structures are donated as modified structure 1 (MS1), modified structure 2 (MS2) and modified structure3 (MS3) in Fig 5(b) to 5(d). MS1 means split in electrical slab that is linked between two symmetrical joint T-D shapes. MS2 means split between T- and D-shape are joint slab indicated by j1. Finally, MS3 means split in electrical slab and split in j1 for both of the Symmetrical T-D structure. MS1 separates the two symmetrical T-D Shape, MS2 separates the two alphabet structure T and D. Finally, MS3 separate two T-, D- joint and two symmetrical T-D joint part.

**Fig 6:**
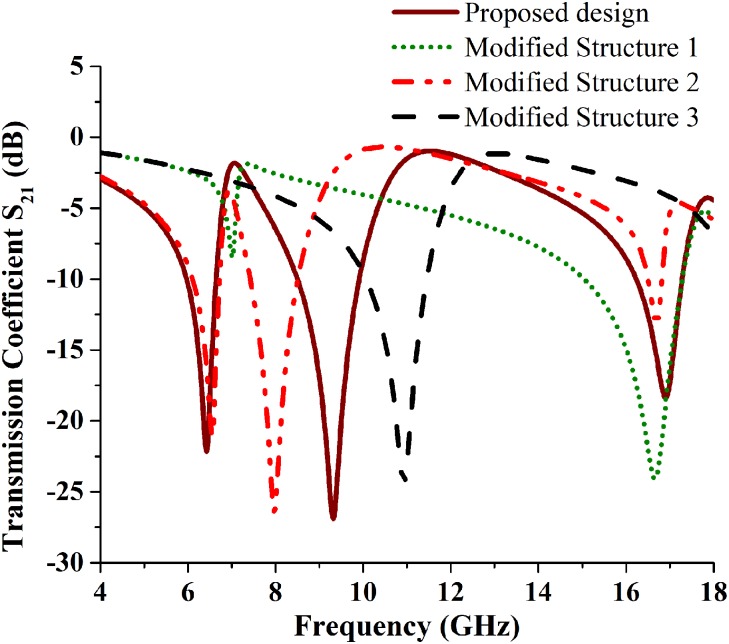
Resonance frequency points of proposed, MS1, MS2 and for MS3 metamaterial structures.

Table 2 and Fig 6, illustrates the resonance frequencies of modified structures (MS1, MS2 and MS3) as well as proposed metamaterial structure. When modified structure 1 is analyzed for its resonance characteristics, then it creates only two resonating points which are denoted by transmission coefficient, but the resonating point in 7.02 GHz with -8.40 dB, which is not valid resonance. Therefore, MS1 has only one resonance frequency of 16.68 GHz, which is only working in Ku band. The MS2 design contains three resonance frequency but its working bands are C-and Ku-band. The MS3 design has also resonance frequency but it's only working in the X-band region. Compare with the proposed metamaterial structure with the modified structures, it can be concluded that the proposed metamaterial has better resonance frequencies and applicable for Tri-band applications.

**Table 2:** Summary of resonance points of proposed and modified metamaterial structures.

Structure type	Resonance Peaks (GHz)	Working band
**Proposed structure**	6.42, 9.32, 16.90	C, X, Ku
**Modified structure 1**	16.68	Ku
**Modified Structure 2**	6.54, 7.98, 16.72	C, Ku
**Modified Structure 3**	10.95	X

**Fig 7:**
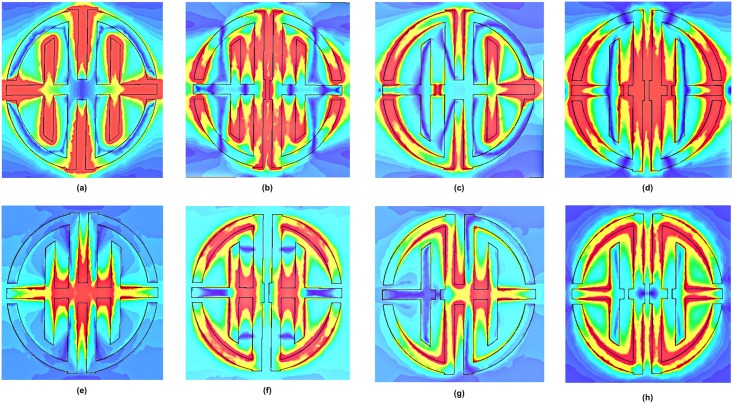
E-fields for (a) proposed structure at 9.32 GHz (b) MS1 at 16.68 GHz (c) MS2 at 7.98 GHz (d) MS3 at 10.95 GHz and H-fields for (e) proposed structure at 9.32 GHz (f) MS1 at 16.68 GHz (g) MS2 at 7.98 GHz (h) MS3 at 10.95 GHz.

Different design structure reacts with electromagnetic field differently. The electric field and magnetic field are differently reacting with metal structure and dispersive material combination. Metal structure and dispersive material combined package respond like an inductor and capacitor based LC resonant circuit. The metal strip is behaving like inductors and its gaps are behaved like a capacitor. Therefore, when metal strip and its gap are changing then inductance and capacitance effects are also changing in the structure. Effect of electric and magnetic field are represented while electric field applied in the x axis and magnetic field applied in the y axis. Fields are behaving differently for increasing the split gap or separate the two symmetric designs. The effect of both fields in structures at resonance frequency is presented in Fig 7. Resonance frequency depends on electromagnetic fields reaction on design structure and fields face different structure they produced a different resonance.

#### Analysis of the design in y-direction

**Fig 8:**
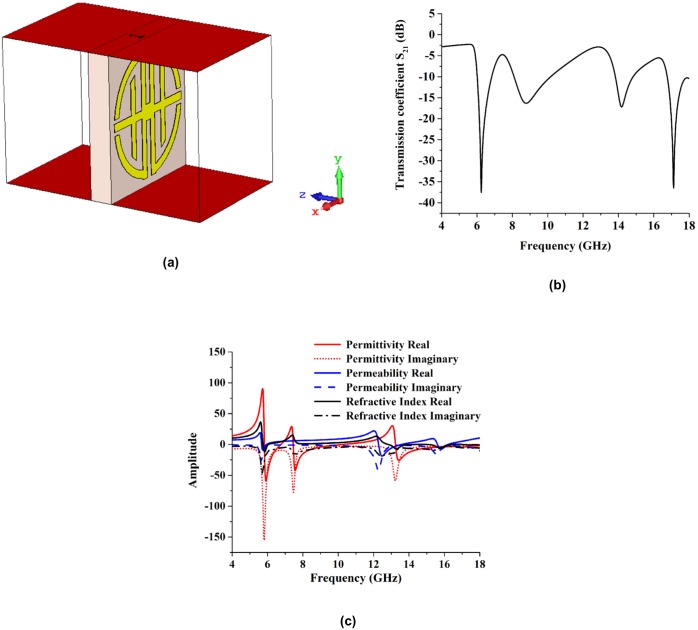
Metamaterial characterization at (a) the geometry for y axis wave propagation, (b) the simulated refraction and transmission coefficient, (c) amplitude of effective parameters.

To establish the biaxial properties electromagnetic waves are propagating in the y-axis direction of the proposed structure. The methodology, the boundary condition and frequency range are same like as z-axis wave propagation. Fig 8(a) shows the geometry of the proposed structure, positioned along the y-axis of electromagnetic wave propagation. Fig 8(b) shows the transmission coefficient S21 of the proposed structure for the y axis wave propagation. From there, the resonance (below -10 dB) frequencies are at 6.05 GHz (-43.77 dB), 8.53 GHz (-16.49 dB), and 13.69 GHz (-40.84 dB). The retrieved values for effective parameter permittivity, permeability, and refractive index are shown in Fig 8(c). From that figure, the negative values of permittivity are 5.83 to 6.74 GHz (bandwidth of 0.91 GHz), 7.45 to 10.23 GHz (bandwidth of 2.78 GHz) and 13.24 to 18 GHz (bandwidth of 4.72 GHz). According to the figure, the negative permeability’s range from 5.68–5.94 GHz and 12.28–13.56 GHz. Additionally, the real value of the negative refractive index ranges from 5.75–6.04 GHz (bandwidth of 0.29 GHz) and 12.84–18 GHz (bandwidth of 5.16 GHz). Here, at 13.36 GHz, the effective parameters of permittivity, permeability, and refractive index are all negative. Therefore, this metamaterial structure is referred to as a left-handed metamaterial.

Table 3 presents a summary of the proposed metamaterial in the y- and z-directions of wave propagation. The biaxial metamaterial has left-handed characteristics in the z-direction, with wave propagation at 11.35 GHz (X-band) and 13.50 GHz (Ku-band). In contrast, in the y-direction, left-handed wave propagation properties occur at 13.36 GHz (Ku-band). From Table 3, the proposed structure represents tuning properties. Both on the direction it produces resonance in the C-band, X band, and Ku-band. For the C-band it was shifted from 6.42 to 6.05 GHz, for the X-band from 9.32 to 8.53 GHz, and for the Ku-band from 16.90 to 13.69 GHz. For all of the cases, the resonance frequencies worked in three applicable bands. Therefore, this is referred to as a biaxial metamaterial with tunable properties.

**Table 3:** Performance of the proposed metamaterial in the z- and y-direction wave propagation.

Propagation Direction	Resonance Frequency (GHz)	Refractive index Bandwidth	Metamaterial Type
z	6.42, 9.32, 16.90	3.55	Left Handed
y	6.05, 8.53, 13.69	5.16	Left Handed

#### Effects of changes to substrate thickness

**Fig 9:**
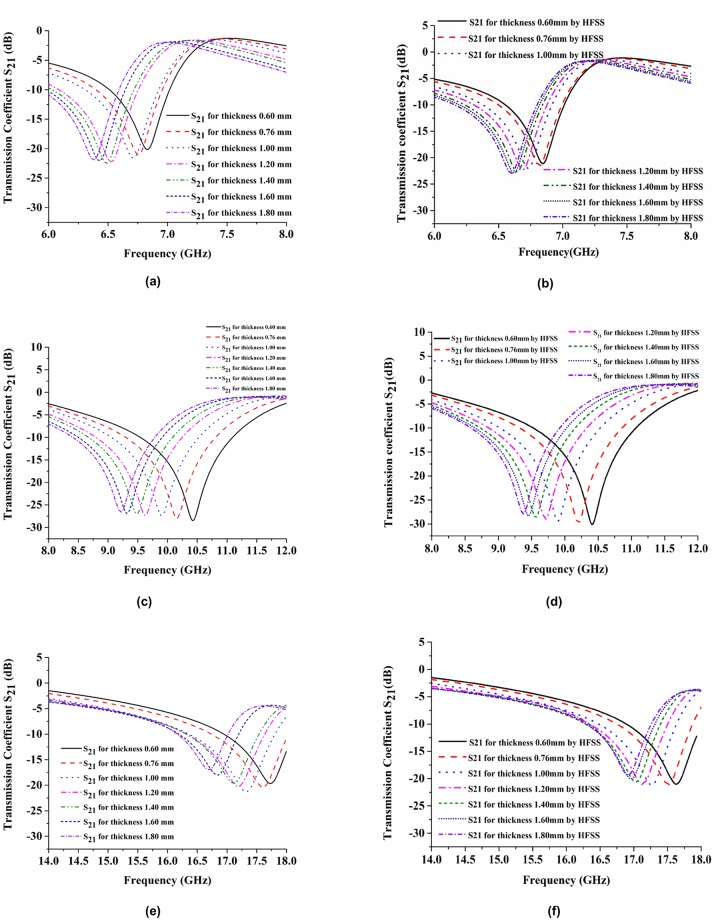
Simulated transmission coefficient at the thickness of 0.50 mm, 0.76 mm, 1.00 mm, 1.20 mm, 1.40 mm, 1.60 mm and 1.60 mm in, (a) C-band, (c) X-band, (e) Ku-band simulated by CST Microwave studio software and (b) C-band, (d) X-band, and (f) Ku-band simulated by HFSS software.

We varied substrate thicknesses and studied how this affected the resonance frequencies and effective electromagnetic properties. Substrate properties like permittivity, permeability, and loss tangent were kept constant, and only the substrate thickness was varied. This was done to verify the tuning properties. Fig 9 shows the simulated S_21_ and effective parameters of the proposed metamaterial for different substrate thicknesses. However, the transmission coefficient is sufficient to analyze how the substrate thickness affected the effective parameters. We simulated how the behavior of the resonant frequency changed in response to changes in substrate thickness using two different types of software: CST Microwave Studio and HFSS. The CST simulator results are shown in Fig 9(a), 9(c) and 9(e), while the HFSS simulator results are shown in Fig 9(b), 9(d) and 9(f). To identify the better sharpness of the transmission coefficient, the multiband resonance frequencies were divided into three different parts. Fig 9(a), 9(c) and 9(e) show the C-band, X-band, and Ku-band resonance frequencies, respectively. The substrate thicknesses were varied as 0.60, 0.76, 1.00, 1.20, 1.40, 1.60, and 1.80 mm. From that figure, it can be seen that when the substrate thickness increases from 0.60 to 1.8 mm, the resonance frequencies drop from 6.83 to 6.36 GHz (C-band), 10.42 to 9.21 GHz (X-band), and 17.72 to 16.68 GHz (Ku-band). The resonance frequency response magnitude (dB) for the C-band increases at a certain thickness limit after that its response magnitude almost like saturated. For the X-band, the resonance frequency responses had approximately the same magnitudes, while for the Ku-band, the response magnitude of the resonance frequency increased with increasing substrate thickness. However, at thickness values greater than 1.2 mm, the resonance frequencies decreased in magnitude. The analytical results from the software are shown in Fig 9(b), 9(d) and 9(f). These results are almost the same as the CST simulation result.

**Fig 10:**
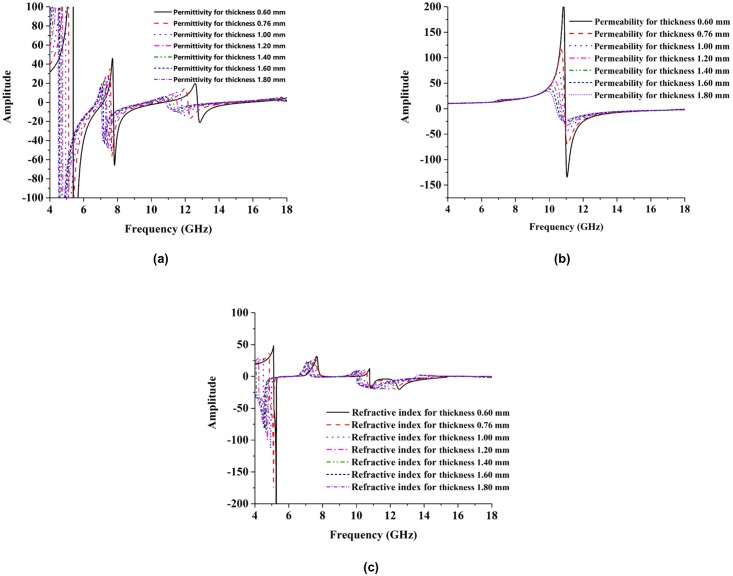
Simulated effective parameters. (a) permittivity (b) permeability and (c) refractive index at the thickness of 0.50 mm, 0.76 mm, 1.00 mm, 1.20 mm, 1.40 mm, 1.60 mm and 1.60 mm.

Fig 10(a) to 10(c) show the effects of substrate thickness variations on the effective parameters. The effects of thickness variations on permittivity are shown in Fig 10(a). From that figure, it can be seen that when the thickness of the substrate increases, the negative permittivity bandwidth also increases. By increasing the substrate thickness, the negative electric response region is shifted from a higher frequency to a lower frequency. In Fig 10(b), the permeability is shown when varying the thickness of the substrate. The negative magnetic response bandwidth is shifted toward a lower frequency by increasing the substrate thickness. The refractive coefficient is shown in Fig 10(c).

In the case of the negative refractive index bandwidth, the substrate thickness when decreases it increases. So this can be summarized that the resonance frequency and effective properties are sensitive to the effective electrical thickness of the substrate. In fact, the joint T-D structure can be treated as an equivalent LC circuit under the excitation of the incident wave. The capacitance increases with the increase in the substrate electrical thickness, therefore the resonance frequencies are reduced. Besides, the field created by the capacitor is limited in the smaller region when the substrate effective electrical thickness increases, therefore the variation of the capacitance develops less and tends to saturate. Table 4 represents the effect of resonance tuning frequencies at different thickness.

**Table 4:** Thickness variation effects on the resonance frequency for proposed metamaterial.

**Thickness (mm)**	0.60	0.76	1.00	1.20	1.40	1.60	1.80
**Resonances (GHz)**	6.83	6.77	6.71	6.52	6.49	6.42	6.36
	10.42	10.16	9.90	9.52	9.48	9.32	9.21
	17.72	17.63	17.32	17.21	17.10	16.90	16.68

### Using nickel aluminate substrate (NiAl_2_O_4_) for metamaterial structure

The electromagnetic wave is propagated on the metamaterial prototype in the z- and y-direction to analyze the biaxial property of the proposed structure in section 4.2.1 and 4.2.2. Later, section 4.2.3 represents the effect of changing thickness substrate.

#### Analyses of design in z-direction

**Fig 11:**
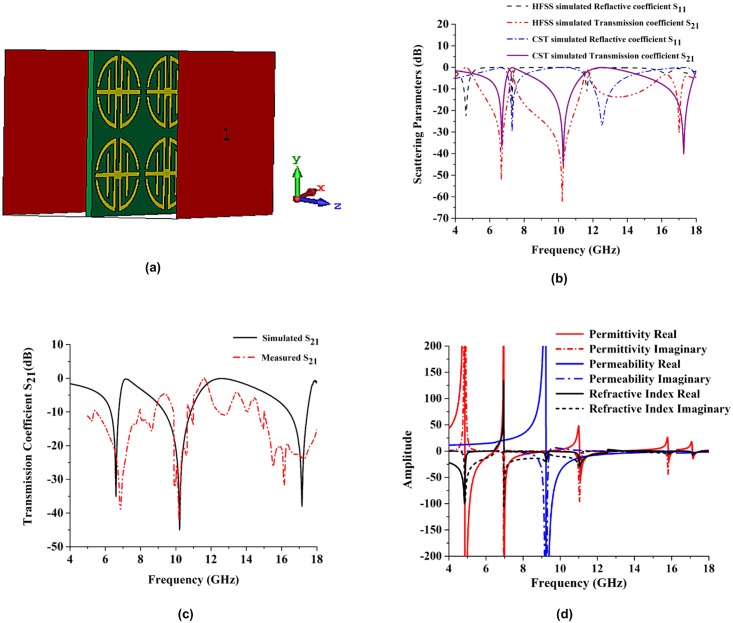
Simulation set-up and experimental results. (a) The geometry for z-axis wave propagation, (b) Reflection and transmission coefficients simulated by CST and HFSS electromagnetic simulator, (c) Measured and simulated transmission coefficients, (d) Amplitude of the effective parameters.

A new flexible nickel aluminate (NiAl_2_O_4_) substrate is used to investigate the metamaterial structure. The electromagnetic wave propagate in the z- direction and its simulated geometry of the proposed metamaterial design present in Fig 11(a). The reflection coefficient (S_11_) and transmission coefficient (S_21_) of the proposed design are also analyzed with two different types of software. The scattering parameters resonance frequency are little bit shifted compared to the FR-4 substrate metamaterial but three resonance frequencies are in same multiband region C-band, X-band and Ku-band. The Resonance frequency has better performance in NiAl_2_O_4_ substrate compare to the FR-4 substrate.

The simulated reflective coefficient (S_11_) and transmission coefficient (S_21_) results are shown in Fig 11(b). These results were obtained from the CST Microwave Studio and HFSS software analyses. The figure shows that the resonance frequencies were 6.70 GHz (C-band and -37.02 dB), 10.27 GHz (X-band and -45.88 dB), and 17.27 GHz (Ku-band and -40.02 dB), which were the simulation results from CST Microwave Studio. The HFSS software simulated resonance frequency was approximately the same as the CST Microwave Studio resonance frequency; however, the resonance dB was much higher in HFSS. The HFSS software results for reflective coefficient and transmission coefficient are shown in Fig 11(b). From that figure, the resonance frequencies are 6.69 GHz (C-band and -52.14 dB), 10.21 GHz (X band and -63.17 dB), and 16.98 GHz (Ku-band and -30.52 dB). The experimental and simulated results for the transmission coefficient of a 4 × 4 array using NiAl_2_O_4_ substrate are shown in Fig 11(c) for the proposed structure. The simulated resonances were 6.58 GHz (C-band), 10.21 GHz, and 17.14 GHz. The measured resonances were 6.85 GHz (C-band), 10.18 GHz (X-band), and 16.17 GHz (Ku-band).

The effective medium parameters for permittivity, permeability, and refractive index are represented in Fig 11(d). The effective permittivity values are negative from 4.99–6.81 GHz (bandwidth of 1.82 GHz), 7.39–10.14 GHz (bandwidth of 2.75 GHz), 12.04–14.59 GHz (bandwidth of 2.55 GHz) and 17.50–17.93 GHz (bandwidth of 0.43 GHz), as shown in Fig 11(d). The effective permeability values were negative from 9.98–18 GHz, as shown in Fig 11(d). Current and applied fields are not in phase in the higher frequency range. However, at lower frequencies, they are in phase with one another, so permeability becomes negative at higher frequencies. Negative refractive index values occur from 4–6.48 GHz (bandwidth of 2.48), 7.42–13.76 GHz (bandwidth of 6.34) and 17.07–18 GHz (bandwidth of 0.30 GHz). The maximum negative refractive index bandwidth is 6.34 GHz. At 17.52, 12.09, and 10.01 GHz, the amplitudes of permittivity, permeability, and refractive index are negative respectively. Therefore, left-handed characteristics are exhibited at 17.52 GHz, 12.09 GHz and 10.01 GHz.

**Fig 12:**
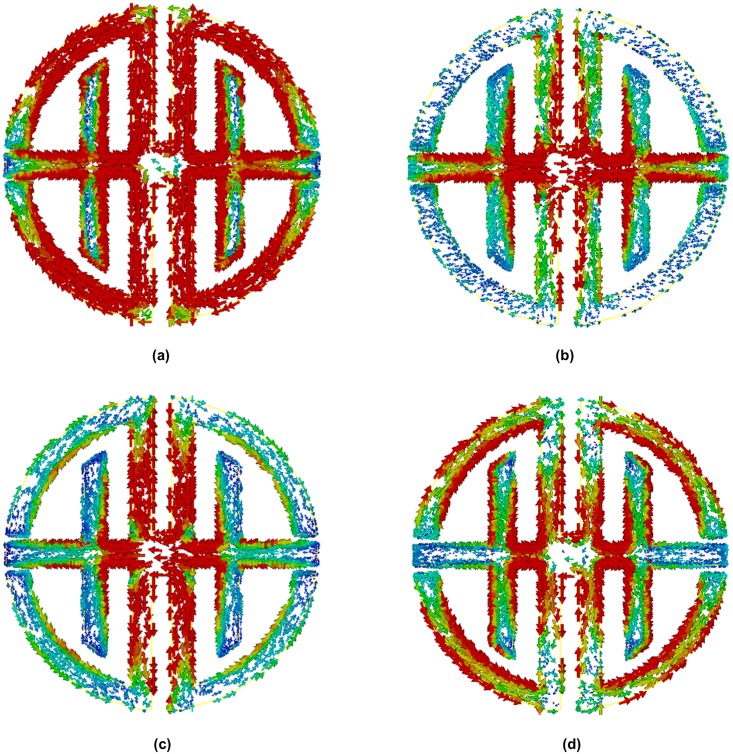
Surface current distribution at (a) 6.85 GHz, (b) 10.18 GHz, (c) 16.17 GHz and (d) 12.09 GHz.

To analyze the physical characteristics of the proposed design, surface current distributions were obtained for different frequencies. The surface current distributions of the proposed unit cell at frequencies of 6.85, 10.18, 16.17, and 12.09 GHz are shown in Fig 12(a) to 12(d). The proposed nickel aluminate substrate metamaterial had a much stronger surface current reaction than the FR-4 substrate metamaterial. At a frequency of 6.85 GHz, the current intensity was much stronger throughout the structure because of the substrate ferromagnetic properties and resonance magnitude. At this frequency, stronger surface currents occur, because of the transmission coefficient bandwidth. In Fig 12(c), the surface current is similar to that in Fig 12(a). However, its current distribution is weaker than the first resonance frequency. At this frequency, the current is stronger at the joint point of the T- and D-shapes. In Fig 12(b) and 12(d), the surface current distributions are more concentrated in the T-shape’s vertical and horizontal part as well as T- and D-shape joint point and D-shape’s vertical part also. The surface currents flow in opposite directions in two opposite ring resonators. Therefore, stop bands are created by nullifying the current.

#### Analysis of the design in y-direction

**Fig 13:**
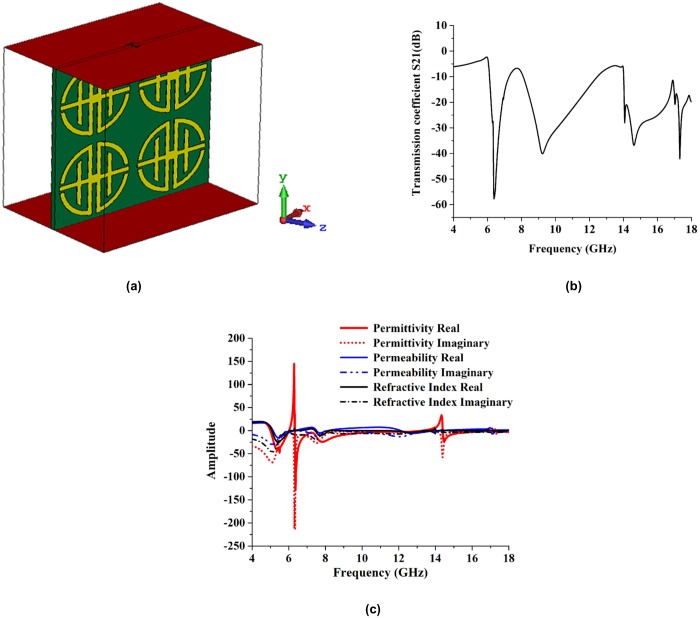
Metamaterial characterization at (a) the geometry at y-axis wave propagation, (b) simulated transmission coefficient, (c) effective permittivity, permeability and refractive index parameters.

The proposed substrate metamaterial structure, then replaced in the y-direction of electromagnetic wave propagation, to demonstrate the biaxial properties. The methodology, boundary conditions, and frequency range were same as for the z-axis wave propagation. Fig 13(a) shows the geometry of the proposed structure, positioned to show y-axis electromagnetic wave propagation. The transmission coefficient of the proposed structure with respect to y-axis wave propagation is shown in Fig 13(b). From the figure, we can see that the resonance frequencies are 6.39 GHz (-58.03 dB), 9.23 GHz (-39.66 dB), 14.62 GHz (-36.79 dB), and 17.30 GHz (-43.01).

The effective parameters of permittivity, permeability, and refractive index are shown in Fig 13(c) for y-axis electromagnetic wave propagation. The negative values of permittivity are 5.00–6.00 GHz (bandwidth of 1 GHz), 6.32–13.29 GHz (bandwidth of 6.97 GHz), 14.40–17.16 GHz (bandwidth of 2.76 GHz) and 17.3–18 GHz (bandwidth of 0.70 GHz) that are shown in Fig 13(c). According to the figure, negative permeability is 5.14–6.33 GHz, 12.10–13.88 GHz, 13.99–14.33 GHz, and 17.14–17.27 GHz. Additionally the real values of the negative refractive index range from 5.09–6.04 GHz (bandwidth of 0.95 GHz), 7.44–8.24 GHz (bandwidth of 0.80 GHz), 8.33–10.34 GHz (bandwidth of 2.01 GHz), 11.25–13.52 GHz (bandwidth of 2.27 GHz), and 15.43–17.90 (bandwidth of 2.47 GHz). The maximum negative refractive index bandwidth is 2.47 GHz. Here, at frequencies of 5.49, 7.82, and 12.45 GHz, all the effective parameters (permittivity, permeability, and refractive index) are negative. Because these frequencies are negative, we can refer to this material as a left-handed metamaterial. Table 5 presents a summary of the proposed metamaterial for the nickel aluminate substrate in the y- and z-directions of electromagnetic wave propagation. The biaxial metamaterial shows left-handed characteristics in the z-direction of wave propagation at 10.01, 12.09, and 17.52 GHz. In contrast, in the y-direction, wave propagation left-handed properties are exhibited at 5.49, 7.82, and 12.45 GHz. From Table 5, we can see that the present structure has tuning properties. Both on the direction it gives resonance in the C-band, X band, and Ku-band. For the C-band, it shifted from 6.58 GHz to 6.39 GHz, for the X-band from 10.21 GHz to 9.23 GHz. In the Ku-band, two resonances were produced for y-axis wave propagation. For both axes, resonance frequencies worked in three bands in a fixed region, and the material can be tuned by changing its electromagnetic wave propagation. Therefore, it is referred to as a biaxial tuned metamaterial.

**Table 5:** Performance of the proposed metamaterial in the z- and y-direction wave propagation.

Propagation Direction	Resonance Frequency (GHz)	Refractive index Bandwidth	Metamaterial Type
z	6.58, 10.21, 17.14	6.34	Left Handed
y	6.39, 9.23, 14.62, 17.30	2.47	Left Handed

#### Effects of changing substrate thicknesses

**Fig 14:**
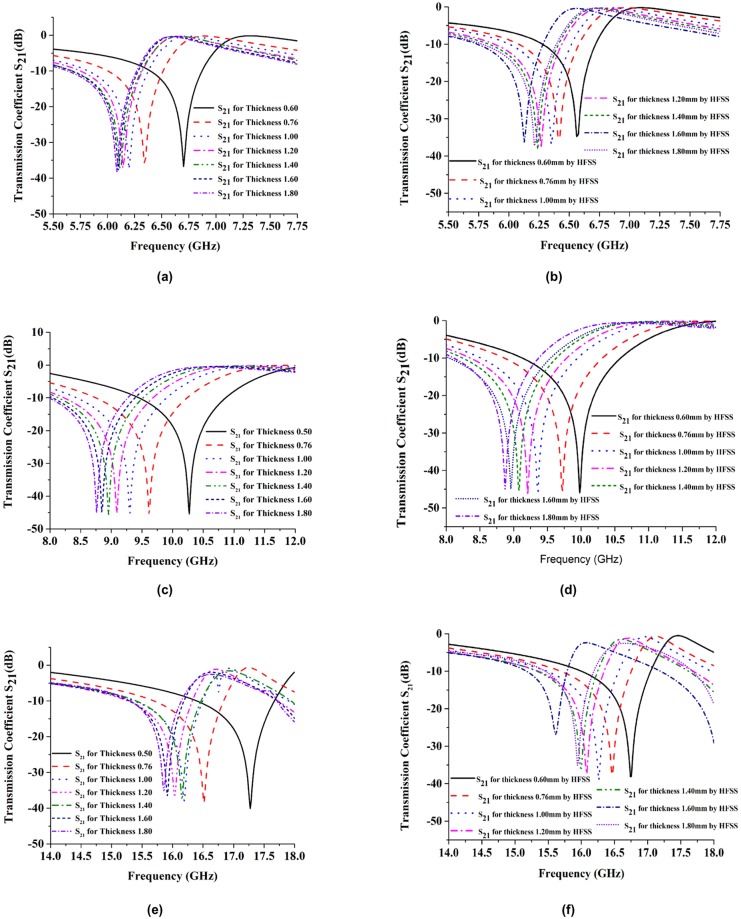
Simulated transmission coefficients at the thickness of 0.50 mm, 0.76 mm, 1.00 mm, 1.20 mm, 1.40 mm, 1.60 mm and 1.60 mm in, (a) C-band, (c) X-band, (e) Ku-band simulated by CST Microwave studio software and (b) C-band, (d) X-band, and (f) Ku-band simulated by HFSS software.

**Fig 15:**
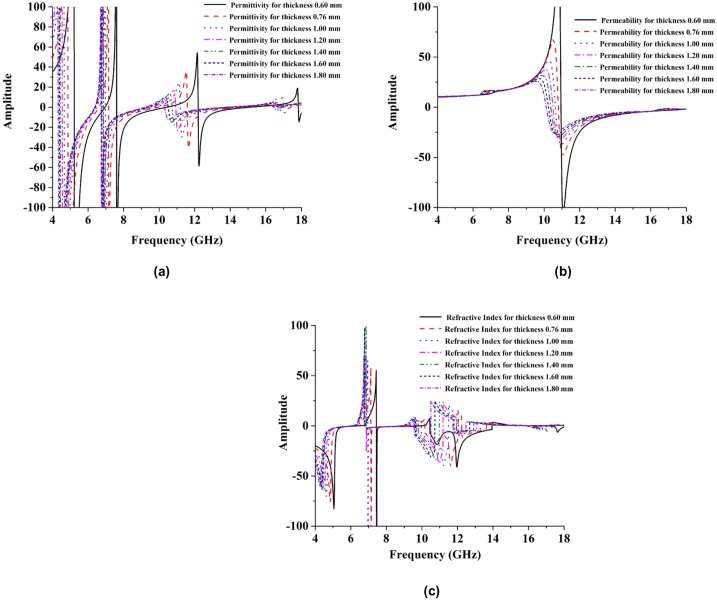
Simulated effective parameters at the thickness of 0.50 mm, 0.76 mm, 1.00 mm, 1.20 mm, 1.40 mm, 1.60 mm and 1.60 mm are, (a) permittivity, (b) permeability and (c) refractive index.

The thickness of the nickel aluminate substrate was also varied, to improve its performance relative to the FR-4 substrate, and examine how its resonance frequency and electromagnetic properties were affected. All of the electromagnetic properties (like, permittivity, permeability, and loss tangent) were kept constant during the thickness variations, to demonstrate the existence of the tuning property. Fig 14 shows how the resonance frequency was affected by variable substrate thicknesses, while Fig 15 shows how the effective parameters were affected by variable substrate thicknesses.

The transmission coefficient (S_21_) values of the proposed structure for different thicknesses are shown in Fig 14(a) to 14(f). The multiband resonance frequency is divided into three different parts to identify the sharpness of the transmission coefficient (S_21_) frequency. Fig 14(a), 14(c) and 14(e) show C-band, X-band and Ku-band resonance frequencies for varying substrate thicknesses (0.50, 0.76, 1.00, 1.20, 1.40, 1.60 and 1.80 mm). Results were simulated with CST Microwave Studio. The resonance frequencies dropped from 6.58 GHz to 6.08 GHz in the C-band regime in Fig 14(a). In Fig 14(c) X-band resonance frequencies are shown for 0.60 to 1.80 mm substrate thicknesses. There, the resonance frequencies dropped from 10.01 to 8.76 GHz in the X-band and in Fig 14(e), a Ku-band resonance frequency sharpness is shown where the resonance frequency dropped from 16.96 to 15.85 GHz. In Fig 14(b), 14(d) and 14(f) the HFSS results for resonance frequencies are shown for various substrate thicknesses.

Notice that the resonance frequencies become saturated with increases in the thickness of the substrate. In Fig 15(a) to 15(c) we show how the effective parameters are affected by variations in the thickness of the substrate. The effects of thickness variations on the permittivity values are shown in Fig 15(a). From that figure, it can be seen that when the thickness increases, the negative electric response region shifts from a higher frequency to a lower frequency. The bandwidth of negative permittivity increases with increasing substrate thickness. In Fig 15(b), the permeability’s are shown for substrates with varying thicknesses. The negative magnetic response region is shifted toward a lower frequency by increasing the substrate thickness. The refractive coefficient is shown in Fig 15(c).

The negative refractive index bandwidth increases with decreasing substrate thickness. A negative refractive index is formed because of the opposite directions of the phase and group velocity. However, it can also be said that the resonance frequency and effective properties are affected by the effective electrical thickness of the substrate. Under the excitation of an incident wave, the proposed structure can also be treated like an equivalent LC resonant circuit. By increasing the electrical thickness of the substrate, the capacitance also increases, just as it does for the FR-4 substrate. As a result, the resonance frequency is reduced or shifted to a low frequency level. Besides, the created capacitance field is limited to a small region while the substrate thickness increases. As a result, the capacitance change become increasingly less saturated for further increases in thickness. Besides, when the substrate thickness increases, its resonance frequency decreases for all applicable bands. The effective properties can be altered significantly by changing the substrate thickness. Table 6 presents the effects of resonance tuning for different thickness values.

**Table 6:** Thickness variation effect on resonance frequency for proposed metamaterial structure.

**Thickness (mm)**	0.60	0.76	1.00	1.20	1.40	1.60	1.80
**Resonances (GHz)**	6.58	6.33	6.19	6.14	6.11	6.10	6.08
	10.01	9.61	9.30	9.09	8.95	8.84	8.76
	16.96	16.51	16.19	16.03	16.15	15.91	15.85

## Comparison between the nickel aluminate (NiAl_2_O_4_) and FR-4 substrate material

Nickel shows ferromagnetic properties. Because of these properties, the nickel aluminate substrate performs better than the FR-4 substrate. When electromagnetic waves propagate over a material, the electric and magnetic fields oscillate with a sinusoidal pattern. The electrical conductivity of any material that actually depends on the internal structure of the material dominates the velocity of the electromagnetic wave running through it. The relative speed of an electrical signal traveling through a material varies according to variations in the material’s internal structure; these variations create different transmission characteristics. The effective parameters are directly related to current distribution and charge properties of the medium. The nickel aluminate substrate resonance point shifts slightly relative to the FR-4 substrate. According to the two software programs and measured data, the obtainable dB magnitude for nickel aluminate is higher than that for the FR-4 substrate metamaterial. The value of the negative refractive index bandwidth is approximately double for the nickel aluminate substrate based metamaterials. The surface current distribution is more concentrated, and higher current flow occurs in the nickel aluminate substrate metamaterial structure, for almost the same resonance and DNG frequency. In both cases, the proposed design exhibits left-handed properties. For thickness variation processes, the thickness of the FR-4 substrate is varied from 0.60 mm to 1.80 mm. On behalf of C-band, X-band and Ku-band, resonance frequency are drops 0.47 GHz, 1.21 GHz and 1.04 GHz bandwidth when the thickness increases. Therefore, the resonance frequency can be tuned by changing the substrate thickness within that range. In contrast, for the nickel aluminate substrate based metamaterial, C-band, X-band and Ku-band resonance frequency are covered 0.50 GHz, 1.34 GHz and 1.11 GHz bandwidth tuning by increasing the substrate thickness. Within that thickness limit, the nickel aluminate metamaterial performed better than the FR-4 metamaterial.

**Fig 16:**
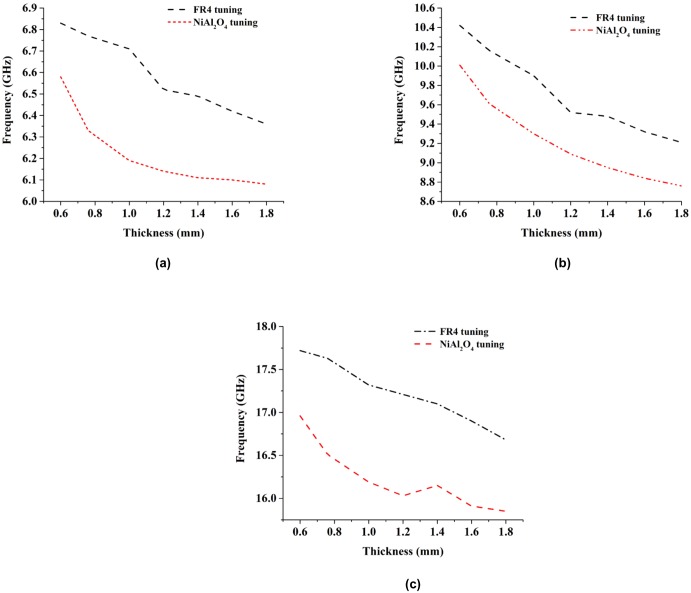
The resonance frequencies are changing with different substrate thickness at (a) C-band (b) X-band (c) Ku-band.

A comparison of how the resonance frequency can be tuned by changing the substrate thickness is shown in Fig 16(a) to 16(c). Three different frequency bands are divided into three parts to produce a more specific result. Fig 16(a) shows that by increasing the thickness of the nickel aluminate substrate, the resonance frequency decreases. However, at a certain point saturation conditions are reached. Further increases in the resonance frequency create little effect under these saturation conditions. Therefore, from that thickness range, the substrate can be chosen for the specific C-band resonance frequency. Similarly, it can be applied to the X-band and Ku-band. The equations used to derive the effect on resonance frequency of increasing the nickel aluminate substrate thickness for the C-band, X-band and Ku-band are:

$$f=-0.7804t^{3}+3.3507t^{2}-4.8911t+8.411$$ (11)

$$f=-0.6801t^{3}+3.255t^{2}-56635t+12.367$$ (12)

$$f=-1.9004t^{3}+7.7049t^{2}-10.537t+20.913$$ (13)

where f and t denote the resonance frequency and substrate thickness, respectively. Eqs (11)–(13) are derived for the thickness variation effect of different substrates on C-band, X-band, and Ku-band applications, respectively. For FR-4 substrates, the effects of thickness variations on resonance frequencies for C-band, X-band, and Ku-band applications are shown in Fig 16(a) to 16(c). Its resonance frequency change rate is less than that of the nickel aluminate substrate. Within the thickness limit from 0.50 to 1.80 mm, the resonance frequency changes at a higher rate at lower thicknesses than it does at higher thicknesses. As a result, changing in the thickness of the FR-4 substrate, it can turn the resonance frequency into saturation. The following equations are derived for the effects of C-band, X-band, and Ku-band application substrate thicknesses on resonance frequency.

$$f=0.297t^{3}-0.9909t^{2}+0.6017t+6.7624$$ (14)

$$f=-0.0289t^{3}+0.6771t^{2}+2.4936t+11.683$$ (15)

$$f=-0.4882t^{3}+1.7425t^{2}-2.7821t+18.893$$ (16)

Where, t and f denotes the substrate thickness and resonance frequency. Eqs (14)–(16) derive from the thickness variation effect of different substrate in C-band, X-band and Ku-band application respectively.

Table 7 compares the operating frequencies, prototype dimensions, frequency bands, metamaterial classifications, and types of substrates used in previous work. The designed metamaterial structure works in C-, X- and Ku-bands, and only for 9 × 9 mm^2^ dimensions, which were tested with FR-4 and NiAl_2_O_4_ substrate materials. The metamaterial structure was fabricated on a flexible nickel aluminate substrate, which had a covered extra-wide band and negative refractive index 6.34 GHz (these were not found in previous work). In both cases, the effective medium ratio of the metamaterial structure is 5, indicating that it is compact in size. Furthermore, [6] used a biaxial left-handed metamaterial that worked in multiband. However, it covered a negative refractive index of only 5.81 GHz. Although [8, 11, 12] were compact in size, they did not work in multiband and had no biaxial properties. In [9], the metamaterial structure worked in tri-bands. However, its size was larger than the proposed metamaterial structure.

**Table 7:** Comparing the experimental results of the proposed structure to the previous work.

Properties	Hasan et al. Ref. [6]	Benosman et al. Ref. [8]	Hasan et al. Ref. [9]	Rizwan et al. Ref. [11]	Dhouibi et al. Ref. [12]	Proposed Design
Dimension (mm^2^)	9×9	3.63×3.63	10×10	2×2	6×6	9×9
Substrate Material	FR-4	FR-4	FR-4	FR-4	FR-4	FR-4 & NiAl_2_O_4_
Working Frequency (GHz)	2 to 14	10×10	2 to 14	1 to 40	4 to 5.5	4 to 18
Bands	C-, X-, Ku	Ku	S-, C-, Ku	K-, Ka	C	C-, X-, Ku
Metamaterial Type	Biaxial Left Handed	Left Handed	Left Handed	Left Handed	Single Handed	Biaxial Left Handed

## Conclusions

A new joint T-D shaped biaxial metamaterial was presented for C-, X- and Ku-band applications. The metamaterial could be tuned by varying the thickness of the substrate material (FR-4 or flexible NiAl_2_O_4_). Based on the structural properties and performance characteristics of these two substrates, we determined that nickel aluminate was a better material than FR-4. The designed structure exhibited a negative maximum refractive index bandwidth for y-directional wave propagation at 5.16 GHz. The thickness of the FR-4 substrate varied from 0.60 to 1.80 mm to assess the tuning effects on resonance frequencies and effective parameters. Because nickel has ferromagnetic properties, the new developed flexible nickel aluminate substrate material performed better than FR-4 substrate for changing substrate thicknesses. Finally, from our results, we confirm that the selected substrate significantly affects the performance of metamaterials.
